# Behavior of Ultrasensitive C-Reactive Protein in Myocardial Revascularization with and without Cardiopulmonary Bypass

**DOI:** 10.21470/1678-9741-2018-0235

**Published:** 2018

**Authors:** Rafael Diniz Abrantes, Alexandre Ciappina Hueb, Whady Hueb, Fabio B. Jatene

**Affiliations:** 1 Cardiovascular Surgery Division, Hospital das Clínicas Samuel Libânio (HCSL), Pouso Alegre, MG, Brazil.; 2 Cardiovascular Surgery Division, Instituto do Coração do Hospital das Clínicas da Faculdade de Medicina da Universidade de São Paulo (InCor-HCFMUSP), São Paulo, SP, Brazil.

**Keywords:** C-Reactive Protein, Atherosclerosis, Myocardial Revascularization, Coronary Artery Bypass, Coronary Artery Bypass, Off-Pump, Inflammation, Cardiopulmonary Bypass

## Abstract

**Objective:**

To analyze the inflammation resulting from myocardial revascularization techniques with and without cardiopulmonary bypass, based on ultrasensitive C-reactive protein (US-CRP) behavior.

**Methods:**

A prospective non-randomized clinical study with 136 patients was performed. Sixty-nine patients were enrolled for Group 1 (on-pump coronary artery bypass - ONCAB) and 67 patients were assigned to Group 2 (off-pump coronary artery bypass - OPCAB). All study participants had blood samples collected for analysis of glucose, triglycerides, creatinine, total cholesterol, high-density lipoprotein (HDL), low-density lipoprotein (LDL) and creatinephosphokinase (CPK) in the preoperative period. The samples of creatinephosphokinase MB (CKMB), troponin I (TnI) and US-CRP were collected in the preoperative period and at 6, 12, 24, 36, 48 and 72 hours after surgery. We also analyzed the preoperative biological variables of each patient (age, smoking, diabetes mellitus, left coronary trunk lesion, body mass index, previous myocardial infarction, myocardial fibrosis). All angiographically documented patients with >70% proximal multiarterial stenosis and ischemia, documented by stress test or classification of stable angina (class II or III), according to the Canadian Cardiovascular Society, were included. Reoperations, combined surgeries, recent acute myocardial infarction, recent inflammatory disease, deep venous thrombosis or recent pulmonary thromboembolism, acute kidney injury or chronic kidney injury were not included.

**Results:**

Correlation values between the US-CRP curve and the ONCAB group, the treatment effect and the analyzed biological variables did not present expressive results. Laboratory variables were evaluated and did not correlate with the applied treatment (*P*>0.05).

**Conclusion:**

The changes in the US-CRP at each moment evaluated from the postoperative period did not show any significance in relation to the surgical technique applied.

**Table:** 

Abbreviations, acronyms & symbols				
**AKI**	**= Acute kidney injury**		**HDL**	**= High-density lipoprotein**
**AMI**	**= Anterior myocardial infarction**		**KF**	**= Kidney failure**
**BMI**	**= Body mass index**		**LCT**	**= Left coronary trunk lesion**
**CABG**	**= Coronary artery bypass grafting**		**LDL**	**= Low-density lipoprotein**
**CAD**	**= Coronary artery disease**		**LVEF**	**= Left ventricular ejection fraction**
**CCS**	**= Canadian Cardiovascular Society**		**MI**	**= Myocardial infarction**
**CKMB**	**= Creatinephosphokinase MB**		**ONCAB**	**= On-pump coronary artery bypass**
**CMR**	**= Cardiac magnetic resonance**		**OPCAB**	**= Off-pump coronary artery bypass**
**CPB**	**= Cardiopulmonary bypass**		**PTE**	**= Pulmonary thromboembolism**
**CPK**	**= Creatinephosphokinase**		**SAH**	**= Systolic arterial hypertension**
**CKI**	**= Chronic kidney injury**		**SIRS**	**= Systemic inflammatory response syndrome**
**CRP**	**= C-reactive protein**		**ST**	**= Stress test**
**CVA**	**= Cerebrovascular accident**		**TC**	**= Total cholesterol**
**CVEs**	**= Cardiovascular events**		**TG**	**= Triglycerides**
**DM**	**= Diabetes mellitus**		**TnI**	**= Troponin I**
**DVT**	**= Deep vein thrombosis**		**US-CRP**	**= Ultrasensitive C-reactive protein**

## INTRODUCTION

In 1930, Tillet and Francis^[1]^ published the first report on the occasional discovery of C-reactive protein (CRP). In 1943, the first clues to the possible connection between CRP and atherothrombotic events were described by Lofstrom^[2]^ and later by Kroop and Shackman^[3]^, in the mid-1950s.

But it was in the mid-1990s, through immunoassays, that this protein with a pentameric structure gained considerable worldwide interest when its prognostic involvement for future cardiovascular events (CVEs) was published^[4]^. Recent studies have shown the central role of inflammation in coronary artery disease (CAD)^[5]^, as well as its influence on the instability of the coronary plaques causing acute CVEs^[6,7]^. This latter characteristic emphasizes the utmost importance for this work in the choice of the ultrasensitive C-reactive protein (US-CRP) for the evaluation of the inflammatory profile resulting from on-pump coronary artery bypass (ONCAB) and off-pump coronary artery bypass (OPCAB).

Even with all the advances achieved in cardiovascular surgery, the circuit used for cardiopulmonary bypass (CPB) still leads to perioperative and postoperative disorders, the most common being the systemic inflammatory response syndrome (SIRS), and coagulation disorders^[8,9]^.

The injuries caused by CPB during the surgical procedure motivated a great deal of interest in recent studies on OPCAB, pioneered by Kolessov^[10]^, in 1964. Following the hypothetical current of CPB withdrawal to minimize the risks of the surgical procedure^[11]^, some initial series of patients undergoing OPCAB were published, with a special nod to Buffolo et al.^[12-14]^, in Brazil, and Benetti et al.^[15]^, in Argentina.

Biomolecular studies have deepened in recent decades, revealing more details of the inflammatory pathophysiology caused to the human body by CPB. This has become a major attempt to intuitively show that OPCAB has greater benefits for patients.

Cochrane database^[16]^, in contrast to the new trend of thought, disclosed its data showing a higher long-term mortality of OPCAB after a systematic review. Large trials such as MASS-III^[17]^, ROOBY^[18]^, DOORS^[19]^, GOPCABE^[20]^ and CORONARY^[21-24]^, which evaluated the comparative results between the techniques with and without CPB, had, as their primary outcomes, mortality, nonfatal myocardial infarction (MI), cerebrovascular accident (CVA) and kidney failure (KF) with a need for dialysis. However, literature does not present reports comparing the inflammatory profile triggered by the two techniques under trial.

With so many studies and results often contradictory, biological markers become increasingly important in trying to explain the impact caused by one or another surgical technique. And this will be the aim of this work: to evaluate systemic inflammation and its effects through the behavior of US-CRP in ONCAB or OPCAB.

## METHODS

**Fig. 1:**
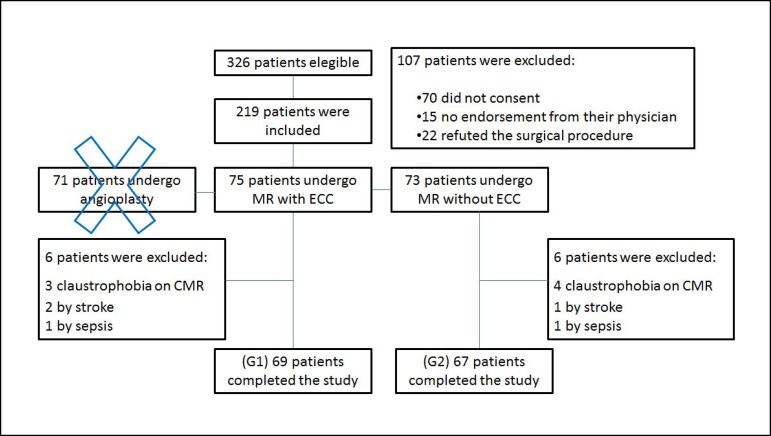
Diagram of MASS-V Trial participants. CMR - Cardiac magnetic resonance.

Source: Modified figure of Hueb et al^[25]^.

Between May 2012 and March 2014, 326 prospective, nonrandomized patients were eligible for coronary artery bypass grafting (CABG) in a single center, and 219 were included in this trial. The main reasons for the exclusion of 107 patients are presented in Figure 1. Of the included patients, 148 were divided into two groups. In Group 1, 75 patients were assigned to undergo ONCAB, and in Group 2, 73 were assigned to undergo OPCAB (Figure 1).

The groups were considered comparable according to the biological and laboratorial variables analyzed, except for the greater occurrence of systolic arterial hypertension (SAH) in Group 1 and acute myocardial infarction (AMI) in Group 2 (Table 1).

**Table 1:** Descriptive values of the evaluated variables.

Variable		Sample (n=136)	Groups		*P*
			With CPB (n=69)	Without CPB (n=67)	
Age (years)		62.19±9.26	61.71±8.60	62.69±9.94	0.541^1^
Age <70 years		104 (76.5%)	54 (78.3%)	50 (74.6%)	0.617^2^
Gender: male		93 (68.4%)	48 (69.6%)	45 (67.2%)	0.763^2^
BMI (kg/m^2^)		28.11±4.34	28.68±4.44	27.53±4.20	0.122^1^
LCT lesion		40 (29.4%)	21 (30,4%)	19 (28.4%)	0.791^2^
Coronary	One	1 (0.7%)	___	1 (1.5%)	
	Two	33 (24.3%)	14 (20.3%)	19 (28.4%)	
	Three	102 (75%)	55 (79.7%)	47 (70.2%)	
	LADA (%)	79.54±16.94	81.61±15.69	77.40±18	0.148^1^
	Cx (%)	77.13±20.03	80.36±16.56	73.46±22.95	0.059^1^
	RC (%)	81.42±21.96	80.74±21.96	82.17±22.11	0.718^1^
Smoking	Yes	36 (26.5%)	23 (33.3%)	13 (19.4%)	
	Ex	74 (54.4%)	37 (53.6%)	37 (55.2%)	
	No	26 (19.1%)	9 (13.1%)	17 (25.4%)	
Prior AMI		43 (31.6%)	16 (23.2%)	27 (40.3%)	0.032^2^
SAH		116 (85.3%)	63 (91.3%)	53 (79.1%)	0.045^2^
DM		68 (50%)	33 (47.8%)	35 (52.2%)	0.607^2^
Angina (degree)	0	16 (11.8%)	8 (11.6%)	8 (11.9%)	
	1	18 (13.2%)	8 (11.6%)	10 (14.9%)	
	2	60 (44.1%)	33 (47.8%)	27 (40.3%)	
	3	28 (20.6%)	12 (17.4%)	16 (23.9%)	
	4	14 (10.3%)	8 (11.6%)	6 (9%)	
Cholesterol		167.47±45.67	162.23±39.36	173.03±51.25	0.176^1^
LDL		97.75±37.27	95.07±34.64	100.60±39.94	0.393^1^
HDL		38.27±12.17	38.51±12	38.02±12.44	0.816^1^
TG		163.64±125.25	154.57±134.34	173.28±115.08	0.390^1^
Glucose		134.04±52.77	134.32±54.28	133.76±51.57	0.951^1^
Creatinine		1.04±0.27	1.05±0.28	1.03±0.26	0.556^1^
Preoperative fibrosis		3.64±5.63	4.42±6.61	2.60±4.80	0.111^4^
Postoperative fibrosis		5.75±6.56	6.16±6.69	5.22±6.42	0.508^4^

AMI=acute myocardial infarction; BMI=body mass index; CPB=cardiopulmonary bypass; Cx=circumflex artery; DM=diabetes mellitus; LADA=left anterior descending artery; LCT=left coronary trunk lesion; RC=right coronary artery; SAH=systolic arterial hypertension; TG=tryglicerides

1 Descriptive level of probability of Student's t-test.

2 Descriptive level of probability of the chi-square test.

3 Descriptive level of probability of the Fisher’s exact test.

4 Descriptive level of probability of the Mann-Whitney non-parametric test.

**Fig. 2:**
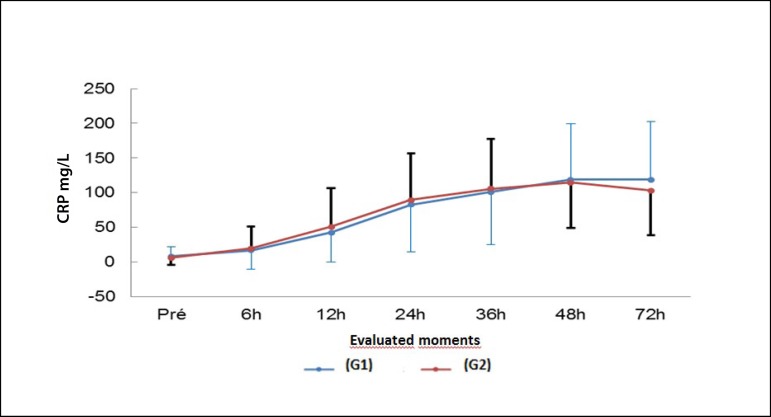
Behavior of the usCRP during the moments evaluated in the groups with and without CPB.

Of all these patients, 12 were excluded (7 for claustrophobia on cardiac magnetic resonance - CMR, 3 for stroke and 2 for sepsis). The remaining 136 patients were divided into 2 groups with 69 patients assigned to Group 1 and 67 to Group 2 (Figure 2). All participants in the study had blood samples collected for the analysis of glucose, triglycerides (TG), creatinine, total cholesterol (TC), high-density lipoprotein (HDL), low-density lipoprotein (LDL) and creatinephosphokinase (CPK) in the preoperative period. The samples of creatinephosphokinase MB (CKMB), troponin I (TnI) and US-CRP were collected in the preoperative period and after 6, 12, 24, 36, 48, and 72 hours from the surgery. The laboratory analysis provided the US-CRP that was analyzed in a univariate and bivariate way. We also analyzed in the preoperative biological variables of each patient [age, smoking, diabetes mellitus (DM), left coronary trunk lesion (LCT), body mass index (BMI), previous MI, myocardial fibrosis]. The presence of myocardial fibrosis was analyzed by CMR 2 days before surgery (F1= preoperative fibrosis) and 6 days after surgery (F2= postoperative fibrosis). All angiographically documented patients with >70% proximal multiarterial stenosis and ischemia, documented by stress test (ST) or classification of stable angina (Class II or III), according to the Canadian Cardiovascular Society (CCS), were included. Reoperations, combined surgeries, recent AMI (≤6 months), recent inflammatory disease, deep vein thrombosis (DVT) or recent pulmonary thromboembolism (PTE), acute kidney injury (AKI), or chronic kidney injury (CKI), were not included.

## RESULTS

It was observed that there was an increase in the US-CRP values obtained in the postoperative period in relation to the preoperative period (*P*<0.001). This change was significant in relation to the myocardial revascularization techniques employed. A bivariate analysis correlated the area under the US-CRP curve and the other variables analyzed and no statistical significance was observed (*P*>0.05), except for the CPK curve that resulted in a positive correlation in Group 1 (*P*=0.015). Figure 2 shows the behavior of the us-CRP at each evaluated moment.

The plasma concentration of US-CRP varied over time in the postoperative period (6h, 12h, 24h, 36, 48h and 72h) and its association with the other variables was assessed by calculating the area below the curve of each patient.

**Fig. 3:**
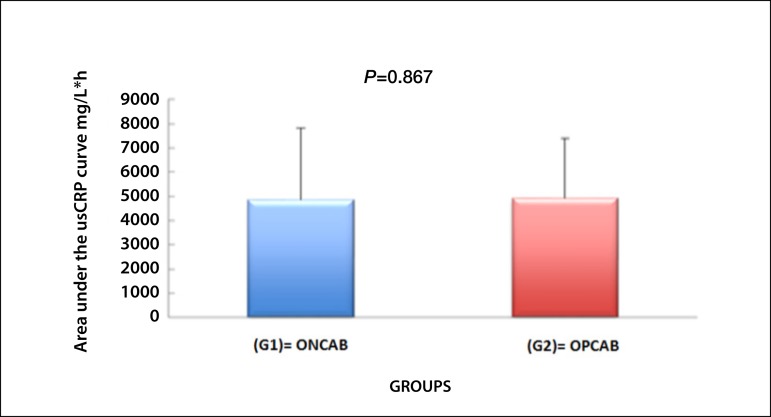
Association of the area under usCRP curve in the studied groups.

The US-CRP of the patients evaluated at each moment did not present statistical difference in the studied groups (*P*=0.867). The means of evaluations in Group 1 and Group 2 are represented in Figure 3.

## DISCUSSION

The results observed in this study were surprising because it is understood that the maintenance of a non-physiological condition, such as CPB, even for a short period, should in some way exacerbate the systemic inflammatory system.

However, it was identified that the use or not of CPB in patients undergoing CABG, being of the same demographic profile, was not the trigger of the inflammatory response identified by US-CRP. It is interesting to observe that the world literature lacks information on US-CRP as a marker of the inflammatory response in the comparison between ONCAB and OPCAB.

The fact is that several well-conducted studies and trials have identified its elevation, but in conditions where there are already predictive variables of elevated or altered systemic injuries^[26]^.

Other studies, such as that of Nezami et al.^[27]^, showed that there was no evolutionary difference in patients submitted to ONCAB or OPCAB, mainly in kidney injury, when US-CRP was assessed.

In this study, we were able to identify that CPB was not the variable that exacerbated this response, at least under the aspect of US-CRP behavior.

This assertion is corroborated by the fact that, in the postoperative period, we identified a marked increase in US-CRP, as shown in the results, specifically in Figure 2, which assesses the US-CRP evolution times.

In the 12^th^ hour, one can observe an increase 40 times greater in the US-CRP baseline. In the 48^th^ postoperative hour, we identified an US-CRP maximum peak, which was almost 100 times the baseline value. However, an interesting point was a similar behavior of US-CRP in patients undergoing ONCAB or OPCAB.

Therefore, we can infer that the inflammatory response was triggered in the postoperative period. In fact, up to 72 hours postoperatively, US-CRP levels still remained very high, but without any difference across the groups. That is, CPB was not the most important variable, as a trigger of the inflammatory response.

In the present study, the idea that the postoperative inflammatory response can be minimized by the non-use of CPB was not supported by the prism of the behavior of US-CRP, which is undoubtedly an important marker of the inflammatory response.

In fact, as pointed out, there are few studies comparing the US-CRP behavior and predictive value in patients undergoing ONCAB or OPCAB. In order to assure the validity of the study, we opted to equalize preoperative demographic variables. Moreover, statistical analyzes segmented the arms according to profiles that could interfere with the results.

Our results were based on univariate and bivariate analyzes regarding the CPB behavior over time and the treatments applied to the groups on or off-pump, thus guaranteeing greater robustness and consistency to the present work.

We observed that, unlike other variables, CPK showed a positive and significant correlation when analyzed with the area of the US-CRP curve in the ONCAB group (*P*=0.015). Therefore, the higher the CPK value, the greater the value of the area under the curve of US-CRP and vice versa. In the OPCAB group we did not observe a significant correlation between these variables (*P*=0.761).

Gerritsen et al.^[28]^ compared patients undergoing ONCAB or OPCAB and identified a worsening of the renal function in patients submitted to ONCAB.

Loef et al.^[29]^ identified signs of increased oxidative stress, as measured by urinary concentrations of hypoxanthine, xanthine, and malondialdehyde in the ONCAB group, while only minor changes were reported in the OPCAB group.

Data derived from the study carried out by Hueb et al.^[25]^, in MASS V, specifically analyzing renal function, did not reveal alterations in renal function when the ONCAB or OPCAB groups were compared.

These insignificant changes in renal function were not connected to inflammatory markers. In our analyzes, we observed that there was no significant correlation between creatinine and the area of the US-CRP curve in the group with or without CPB (*P*=0.797).

Another very relevant aspect in the postoperative CABG is related to the injury that may develop in the myocardium. In this sense, we were careful to analyze the behavior of the myocardial necrosis markers together with US-CRP in the postoperative period.

We analyzed the area under the US-CRP curve and the peak plasma concentration of CKMB and troponin (I) and there was no difference in these biomarkers in the postoperative period, either in Group 1 or in Group 2. Many inflammatory triggers could influence the US-CRP behavior.

In the attempt to avoid bias, there was great concern in the identification and influence of biological variables (age, smoking, BMI, LCT, previous AMI, myocardial fibrosis) and laboratory variables (glucose, TG, creatinine, TC, HDL, LDL, CPK, CKMB, TnI) with pro-inflammatory potential in the behavior of this acute phase inflammatory protein in both techniques employed.

Studies have shown small individual variations in serum concentrations of US-CRP in different, yet very similar, age groups, between men and women^[26-28]^. Older individuals tend to have greater stability in US-CRP blood levels^[29]^.

The two-way analysis of variance, contrary to our expectations, showed no correlation between the area under the US-CRP curve and the subgroups analyzed (*P*=0.127) or the applied treatment (*P*=0.207). There was no prevalence of one myocardial revascularization technique over the other in this study, despite the inflammatory profile of smokers and former smokers.

The strong link between AMI and serum levels of US-CRP is well demonstrated in the literature^[30]^. US-CRP has been shown to be a good predictor of recurrence of new coronary events in patients who have already suffered a heart attack^[31]^. Significantly, more patients with previous AMI were allocated to Group 2 (Table 1).

Regarding this finding, there was no difference in the mean left ventricular ejection fraction (LVEF) between Group 1 (63%) and Group 2 (62%). Hypothetically, there was a bias trend of results due to the inflammatory profile of Group 2 patients.

In contrast to the previous hypothesis, after the correlation between the area under the US-CRP curve and the previous AMI variable, no interaction was observed in the ONCAB group or in relation to the effect of the treatment in face of the CABG techniques employed.

This study showed that there was no preferential CABG technique for patients with AMI, when the US-CRP behavior was analyzed.

We can conclude this discussion by stating that, in relation to the known demographic profile and variables, to predict a worse postoperative prognosis in patients submitted to ONCAB or OPCAB, this study, through the analysis of a reliable inflammatory response marker, revealed that both myocardium revascularization surgery techniques, on or off-pump, promote an increase in the inflammatory response, increasing preoperative to postoperative US-CRP values.

## CONCLUSION

There was an increase in US-CRP in the postoperative period compared to the preoperative period. This increase occurred in all moments assessed postoperatively. There was no difference in the US-CRP behavior between the two myocardial revascularization techniques employed. We inferred that there was an increase the inflammatory process based on the behavior of the US-CRP from the preoperative to the postoperative period, without evidence of correlation with the biological variables (except CPK in the ONCAB group) and the operative techniques employed.

**Table:** 

**Authors’ roles & responsibilities**	
RDA	Substantial contributions to the conception or design of the work; or the acquisition, analysis, or interpretation of data for the work; final approval of the version to be published
ACH	Substantial contributions to the conception or design of the work; or the acquisition, analysis, or interpretation of data for the work; final approval of the version to be published
WH	Substantial contributions to the conception or design of the work; or the acquisition, analysis, or interpretation of data for the work; final approval of the version to be published
FBJ	Drafting the work or revising it critically for important intellectual content; final approval of the version to be published
